# Complete genome sequence of a potentially probiotic cheese isolate *Lactiplantibacillus plantarum* LP140 from the Nordbiotic collection

**DOI:** 10.1128/mra.00606-24

**Published:** 2024-10-08

**Authors:** Agnieszka Szczepankowska, Bożena Cukrowska, Tamara Aleksandrzak-Piekarczyk

**Affiliations:** 1 Institute of Biochemistry and Biophysics, Polish Academy of Sciences, Warsaw, Poland; 2 Department of Pathomorphology, The Children Memorial Health Institute, Warsaw, Poland; SUNY College of Environmental Science and Forestry, Syracuse, New York, USA

**Keywords:** probiotics, lactic acid bacteria, *Lactiplantibacillu*s, genome sequencing, probiogenomics, GABA, polyamines, bacteriocins, genome annotation, cheese isolate

## Abstract

We report the complete genome sequence of *Lactiplantibacillus plantarum* LP140, a cheese isolate from the Nordbiotic collection, comprising 3,371,266 bp with 44.4% GC content. Our data provide insight into the potential of LP140 for use as a probiotic strain.

## ANNOUNCEMENT

*Lactiplantibacillus plantarum* belongs to gram-positive lactic acid bacteria, which are used as starter strains for fermented foods as well as probiotics, providing beneficial health effects to consumers (1–4). *L. plantarum* LP140, originally isolated from cheese, was acquired by Nordic Biotic LTD. who made a deposit at the Leibniz Institute DSMZ-German Collection of Microorganisms and Cell Cultures GmbH (DSM33804). Genomic sequencing and probiogenomic analysis of LP140 revealed genetic markers for the synthesis of bacteriocins, γ-aminobutyric acid (GABA), and taurine.

LP140 was retrieved from the Nordbiotic collection and cultured from a single colony in De Man-Rogosa-Sharpe (MRS) medium (Oxoid) at 37°C overnight under aerobic conditions. Genomic DNA was extracted using the cetyltrimethylammonium bromide/lysozyme method (5) from bacterial cells pretreated with lysozyme (20 mg/mL; Sigma-Aldrich) and mutanolysin (5 U/mL; A&A Biotechnology) (15 min, 37°C).

Genomic DNA sequencing was performed using a hybrid approach, combining high-throughput sequencing on the Illumina MiSeq platform and third-generation sequencing on the Oxford Nanopore GridION. The NEB Ultra II F kit (New England Biolabs) was used for shearing and constructing the genomic DNA library. Illumina MiSeq sequencing was performed with the MiSeq v3 600-cycle chemistry kit cassette, yielding 352,375 quality-filtered 2 × 300-bp paired reads and 163,360,242 nt of sequence. The Oxford Nanopore GridION sequencing library was constructed using the 1D Ligation Kit (LSK109) and encoded using ONT Native barcodes. Sequencing was performed using Flow Cell R9.4.1 on GridION, generating 81,930 reads and 408,021,658 nt sequences with an *N*_50_ value of 6,432. All software was used at default parameters.

Oxford Nanopore sequence reads were assembled, circularized, and rotated using Trycycler (v.0.5.3) (6). Genomic sequences were corrected with Illumina data using Polypolish (v.0.5.0) (7) and Polca (v.4.0.5) (8).

Genome annotation was done using the NCBI Prokaryotic Genome Annotation Pipeline (v.6.6) (9). The genome comprises a single circular closed chromosome and five circular plasmids (Table 1), with a total of 3,106 predicted coding sequences (CDSs), 74 tRNAs, 16 rRNAs, and 4 ncRNAs.

**TABLE 1:** *L. plantarum* LP140 sequencing data

Contig	Size (kb)	%GC	CDS	Illumina coverage	ONT coverage
LP140 (chromosome)	3,206,549	44.7	2,932	44×	105×
pLP140_p1 (plasmid)	61,132	41.3	56	91×	175×
pLP140_p2 (plasmid)	40,748	39.8	39	143×	294×
pLP140_p3 (plasmid)	39,617	41.5	54	88×	131×
pLP140_p4 (plasmid)	14,068	36.8	13	273×	671×
pLP140_p5 (plasmid)	9,152	36.3	12	176×	438×

Probiogenomic analyses revealed genes encoding bacteriocins homologous to the class IIb two-peptide plantaricins JK and EF, as well as the putative bacteriocin plantaricin N and the bacteriocin-like pheromone plantaricin A (10, 11). These findings suggest the potential prohealth properties of LP140 and its role in managing gut dysbiosis (12). Additionally, LP140 carries bile salt hydrolase (BSH) and glutamate decarboxylase (GAD) genes, indicating its capability for bile acid deconjugation and taurine synthesis. The coupled activity of BSH and bile acid-binding taurine may contribute to lowering blood cholesterol levels (13). GAD is also involved in the conversion of L-glutamate into the well-known GABA neurotransmitter (14). GABA produced by the gut microbiota may contribute to health benefits in humans through the gut–brain axis and act as an anti-inflammatory, antioxidant, immunomodulatory, and cholesterol-lowering agent (15, 16).

## Data Availability

Complete sequencing data have been deposited in GenBank under BioProject PRJNA1070959; BioSample SAMN39639084. The whole genomic data are available under accession numbers CP144144 (chromosome), CP144145 (pLP140_p1), CP144146 (pLP140_p2), CP144147 (pLP140_p3), CP144148 (pLP140_p4), and CP144149 (pLP140_p5). Illumina SRA reads are available under accession number SRX23451764. Oxford Nanopore SRA reads are available under acc. no. SRX23451763.
